# Association of the rs1042522 SNP with prostate cancer risk: a study of cancer tissues, primary tumor cultures, and serum samples from a Spanish Caucasian population

**DOI:** 10.3389/fonc.2024.1398411

**Published:** 2024-08-13

**Authors:** Emily Toscano-Guerra, Valentina Maggio, Javier García, Maria Eugenia Semidey, Ana Celma, Juan Morote, Inés de Torres, Marina Giralt, Roser Ferrer-Costa, Rosanna Paciucci

**Affiliations:** ^1^ Cell Signaling and Cancer Progression Laboratory, Vall d’Hebron Institute of Research (VHIR), Barcelona, Spain; ^2^ Department of Biochemistry and Molecular Biology, Autonomous University of Barcelona, Barcelona, Spain; ^3^ Clinical Biochemistry Department, Biochemistry Service, Vall d’Hebron Hospital, Barcelona, Spain; ^4^ Facultad Ciencias e Ingeniería, Universidad Peruana Cayetano Heredia, Lima, Peru; ^5^ Department of Pathology, Vall d’Hebron Hospital, Barcelona, Spain; ^6^ Department of Urology, Vall d’Hebron Hospital, Barcelona, Spain

**Keywords:** TP53, SNP, rs1042522, prostate cancer, European population

## Abstract

**Background:**

Prostate cancer (PCa) is a leading cause of cancer-related deaths in European men, emphasizing the urgent need for effective risk assessment strategies. The TP53 gene, a tumor suppressor gene frequently mutated in cancer, commonly harbors the rs1042522 single nucleotide polymorphism (SNP), known as the P72R SNP, which may influence PCa susceptibility. This study investigated the prevalence of the P72R SNP in European Caucasian PCa samples and its association with PCa risk.

**Methods:**

Genotyping was conducted on 12 hormone-naïve aggressive PCa cultures (hnPCs) from untreated patients (Gleason ≥8), 11 radical prostatectomies (RP), and 94 serum samples using DNA Sanger sequencing and melting curve analysis. Comparative analysis utilized data from the GnomAD database’s European Caucasian non-cancer population.

**Results:**

Our results demonstrate a significantly higher frequency of the P72R SNP in PCa samples and serums compared to the general European non-cancer population. A robust and statistically significant association (p < 0.0001) between the SNP and prostate cancer risk was identified, with an odds ratio of 7.937 (95% CI 5.37-11.00). Notably, the G allele (R72) showed a pronounced prevalence in high Gleason score (≥8) patients, although statistical significance was not reached. These results highlight a potential association with undifferentiated and malignant PCa lesions.

**Conclusion:**

The compelling association between the P72R SNP and prostate cancer risk underscores the potential utility of this marker for the early identification of patients at risk of aggressive metastatic prostate cancer. This insight could empower further research to intervene at an early stage by offering enhanced opportunities for timely and targeted interventions.

## Introduction

Prostate cancer (PCa), which is highly prevalent in developed countries, is the most common cancer in men and the third most common cause of cancer-related deaths in Europe after lung and colorectal cancer (1, 2). In the majority of cases, cancer is efficiently eradicated and cured by surgical prostatectomy, but late-diagnosed or metastatic aggressive cancers have a five-year relative survival rate of less than 30% (3). The tumor suppressor gene *TP53* plays a pivotal role in the prevention of tumor development. Not surprisingly, it has also been described as the most commonly mutated gene in human cancers (4). Somatic *TP53* mutations occur more frequently as later events and are drivers of aggressive and metastatic cancers, including PCa (5).

Several hereditary genetic variations, found in approximately 5-20% of different cancer types, are associated with pathogenic variants of genes, such as *BRCA2* and *ATM*. In PCa, pathogenic germline variants of the *TP53* gene have been described with a relative risk of 4.7-8.6, which is comparable to the frequencies described for those genes (6).

Germline variant alleles, known as single-nucleotide polymorphisms (SNPs), are present in over 1% of the population and occur naturally. Although many of these variants are considered to have minor or not impact on human health, recent significant studies indicate otherwise, particularly in PCa (7, 8). Some common SNPs can indeed affect the structure and function of proteins, thereby contributing to severe health outcomes. In PCa, specific SNPs, such as those in the *BTNL2* gene, have been associated with elevated prostate cancer risk, and structural variants have been linked to poor clinical outcomes and disease progression (9, 10).

Despite significant progress in understanding PCa genetics, the understanding of some controversial germline genetic variations is still incomplete. In particular, the P72R single nucleotide polymorphism (rs1042522) has shown conflicting results in its association with prostate cancer risk and aggressiveness in various reports (11–14). These studies suggest that P72R may influence PCa susceptibility, although the exact nature of this influence remains debated within the scientific community.

By investigating the association of the rs1042522 SNP of the *TP53* gene in cancer tissues, primary tumor cultures, and serum samples, our study aims to provide additional evidence of the association of this SNP with prostate cancer risk in a European Caucasian population. Here, we describe the identification of the rs1042522 in the majority of hnPCs analyzed. Its presence was confirmed in a cohort of 11 radical prostatectomy (RP) tissues and 94 serum samples from Caucasian patients with prostate cancer. The association between the variant allele and prostate cancer risk was also investigated by comparing with the healthy (non-cancerous) European (non-Finnish) population from GnomAD v2.1.1.

These results highlight the potential value of rs1042522 assessment for identifying patients at risk for aggressive metastatic prostate cancer.

## Materials and methods

### Prostate primary culture from hormone-naïve patients

Primary cultures from prostate tumor needle biopsies of hormone-naïve patients (i.e. without previous treatment) were established in our laboratory from a cohort of patients with aggressive/metastatic prostate cancer, selected for (i) high levels of serum PSA (≥50 ng/mL), (ii) positive digital rectal examination (DRE), and (iii) Gleason ≥ 8. hnPCs were cultured at 37°C in an atmosphere of 5% CO2 with complete DMEM-F12 medium containing 2 mM L-glutamine, 100 U of penicillin/mL, 100 µg streptomycin/mL, 0.1 mM nonessential amino acids, 1 mM sodium pyruvate and 7% fetal bovine serum, Supplement 1X, human FGF-10 (10 ng/µL), human EGF (20 ng/µL), and vitamin A and E (200 ng/µL). Growth factors and vitamins were added freshly, and supplements were prepared and stored at -20°C until use. The supplement (100X) was prepared in DMEM-F12 containing glucose (6 mg/mL), transferrin (1000 µg/mL), human insulin (2.500 µg/mL), putrescine (97 µg/mL), sodium selenite (30 µM), and hydrocortisone (100 µM).

### Radical prostatectomy samples

Eleven radical prostatectomy (RP) samples from patients with localized operable tumors (Gleason score ≤7) were compared with the hnPCs’s. Samples were collected by the Service of Urology of the Hospital Vall d´Hebron (HVH), preserved in optimal cutting temperature (OCT) compound, and stored at -80°C until frozen sectioning.

### Serum samples from PCa patients

A cohort of 94 surplus serum samples from Southern European Spanish male patients followed up at the HVH Biochemistry Service, diagnosed with PCa by tissue biopsy and elevated PSA, were randomly selected according to biobank availability, sample quality (non-hemolysed) and ethnicity. Blood samples were collected in SST yellow tubes containing separator gels were centrifuged at 3,500 rpm for 15 min at 4°C. The resulting serum was collected in 2 mL tubes, properly labelled and codified in the biobanking system, and subsequently stored at -80°C.

The study was reviewed and approved by the HVH Institutional Review Board (Medical Research Ethics Committee, protocol number PR(AG) 96/2015).

### DNA sequencing

The variant rs1042522 of the *TP53* gene was first identified in hnPCs and RPs by analysis of complementary DNA (cDNA) using RNA reverse transcription (RT) and Sanger sequencing. Briefly, Total RNA from cells/tissues was isolated using RNeasy mini kit (QIAGEN) according to the manufacturer’s protocol. For tissue samples, a combination of Phenol-based RNA isolation and purification using silica method was used. Good RNA quality was confirmed using a 2100 Bioanalyzer (Agilent), and samples with RIN≥7 were used. cDNA was synthesized from 1.0 µg (cells/tissues) of total RNA using the NZY M-MuLV First Strand cDNA Synthesis Kit (NZY Tech) and 2720 Thermocycler (Applied Biosystems). The resulting amplicons were sequenced using the Sanger method (Macrogen Europe service).

### DNA genotyping

Genomic DNA from hnPCs and RPs was extracted using a DNeasy Mini Kit (Qiagen, Spain), following the manufacturer’s guidelines. For genomic DNA extraction from serum samples, the MagNA Pure 24 Instrument and total NA Isolation Kit (Roche) were used, a fully automated system based on magnetic glass particle technology (MGP) that allows the processing of up to 24 samples in approximately 70 min. Genotyping of the P72R SNP of *TP53* was conducted for all samples by melting curve analysis with a customized LightSNiP assay (TIB MOLBIOL) on a LightCycler 480 II instrument (Roche). Reactions were carried out in a final volume of 20 μL, comprising 2.0 μL of LightCycler FastStart DNA Master HybProbe mix (Roche), 1.0 μL of LightSNiP mix, 3 mM MgCl2, and 50 ng of DNA. The ThermoCycler was set up under the conditions outlined in **Supplementary Table S1**. Subsequently, the melting curves were assessed using the Melt Curve Genotyping software (Roche).

### Control group

To study the association of the P72R SNP with prostate cancer, the frequencies found in the (non-cancer) European (non-Finnish) population from GnomAD v2.1.1, containing 134,187 samples, were used as a control group. In addition, we studied the Spanish Iberian Population from 1000 genomes Project (www.ensembl.org) containing 214 samples.

### Gleason score and grade groups

The Gleason score classification system assesses the aggressiveness of prostate tumors based on the architecture of the cancerous tissues and the degree of cell differentiation, as observed under a microscope. This score, which ranges from 6 to 10, is the sum of two histological patterns (the most common plus the highest grade) within a single tissue sample (**Supplementary Table S2**). To better understand and describe cancer aggressiveness, the Gleason score was adjusted to a grade group encompassing values ranging from one to five. The correlation between the scores and their respective groups is shown in **Supplementary Table 2**.

### Statistics

Associations between frequencies and proportions were analyzed using contingency tables and Fisher’s test. Values were considered statistically significant at *P* < 0.05. These analyses were conducted using the GraphPad Prism 9 software. Data and image analyses were performed using Circos software (http://circos.ca/).

## Results

### The P72R SNP frequency in primary prostate tumour cultures and cancer tissues

**Figure 1:**
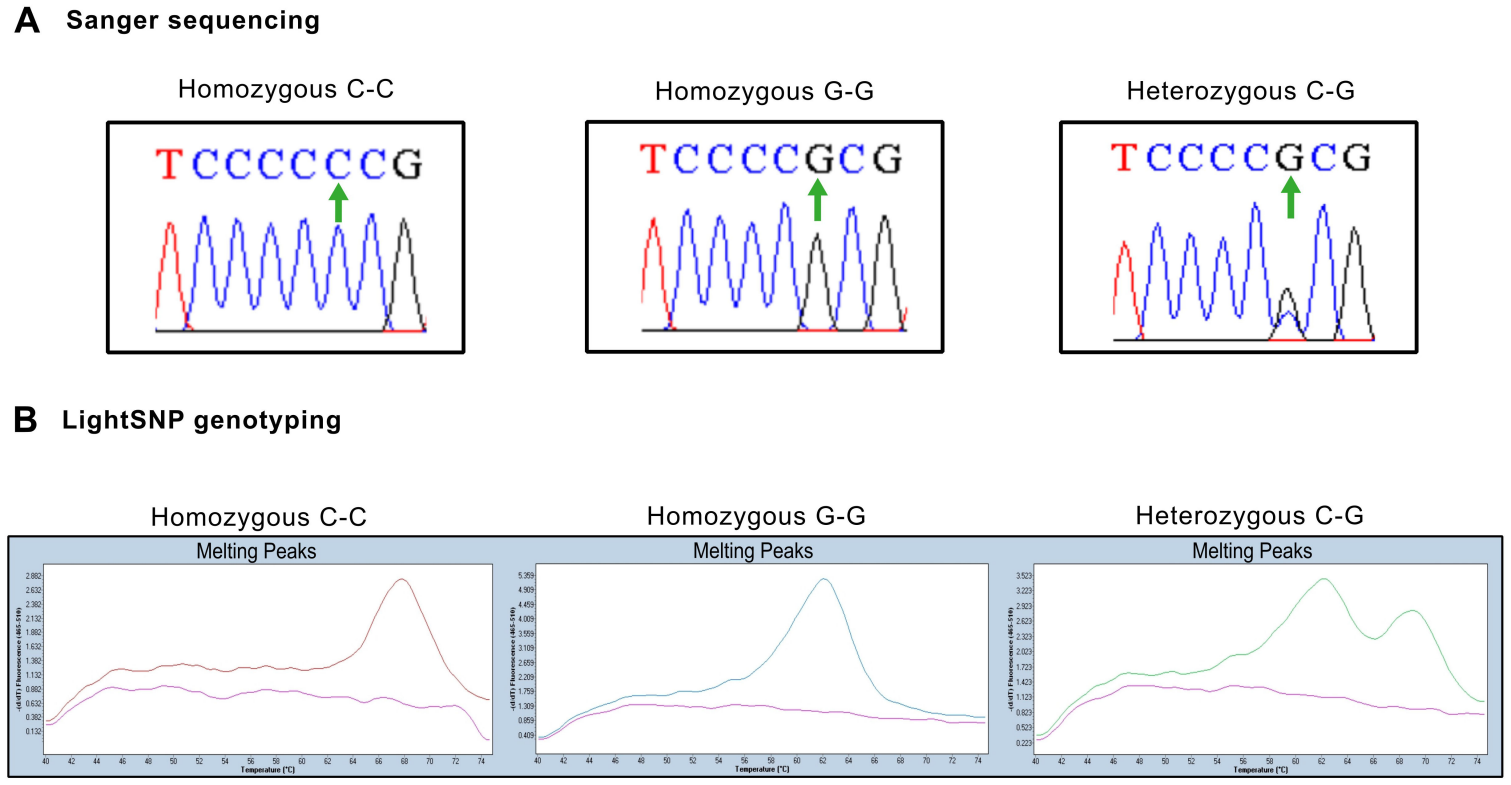
Representative genotyping results for serum and primary culture samples **(A)** Representative Sanger sequencing chromatograms of homozygous and heterozygous samples. Green arrows indicate position 357 in the coding sequence. **(B)** Representative melting curves from serum samples illustrating peaks of absorbance at different dissociation temperatures. Left panel: Homozygous samples with cytosine in both strands show a dissociation peak at 68°C. Center panel: Homozygous samples with guanine in both strands show a dissociation peak at 60°C. Right panel: Heterozygous samples with cytosine and guanine residues are identified by both peaks.

More than 20 different SNPs, have been described in the *TP53* gene (15) including the rs1042522 or P72R variant, at amino acid 72 of p53 (**Supplementary Figure S1A**). This variant contains a guanine at position 357 (C **G** C) of the coding region sequence, which changes the encoded amino acid from proline (C**C**C) to arginine (R or ARG). (**Supplementary Figure S1B**). **Supplementary Figure S1C** shows that arginine induced a decrease in hydrophobicity (turquoise bars) relative to the most abundant proline (P or PRO), which may affect the interaction of the p53 protein with other ligands (CCAR2 or HRMT1L2). To study the potential role of this *TP53* variant in the aggressiveness of unresectable prostate tumors, we examined 12 primary tumor cultures from hormone-naïve patients (hnPCs). Sanger sequencing revealed the P72R SNP (ARG variant) at a frequency of 82.0% (9/11), whereas the PRO variant frequency was 18.0% (2/11), and the heterozygote samples predominantly expressed ARG (**Table 1**, **Figure 1A**). To confirm these results, the hnPCs and, additionally, 11 prostatectomy tissue samples obtained from different PCa patients, were genotyped using gDNA melting curve analysis (**Table 1**, **Figure 1B**). In hnPCs, two homozygotes for cytosine (C, PRO), six homozygotes for guanine (G, ARG), and four heterozygotes were found. In the RP tissues, four homozygotes for cytosine, five homozygotes for guanine, and two heterozygotes predominantly expressed ARG. The total frequencies of C and G in primary tumor cultures were 0.33 and 0.67, respectively, while in RP tissues, the frequency of C was 0.45, and that of G was 0.55, indicating that primary tumor cultures have a greater incidence of the P72R allele than tumor tissues from RP, suggesting that the presence of this SNP may help to distinguish inoperable aggressive cancers from the non-metastatic, less aggressive ones that can be cured by surgery.

**Table 1:** P72R genotyping in primary cultures and radical prostatectomy tissues.

	Sanger sequencing		LightSNiP assay	
SAMPLE	cDNA	Expressed Aminoacid	gDNA	
hnPC01	C **C** C	**Proline**	**CC**	Homozygous Proline
hnPC06	C **C** C	**Proline**	**CC**	
hnPC02	C **G** C	**Arginine**	**GG**	Homozygous Arginine
hnPC03	C **G** C	**Arginine**	**GG**	
hnPC08	C **G** C	**Arginine**	**GG**	
hnPC09	C **G** C	**Arginine**	**GG**	
hnPC10	C **G** C	**Arginine**	**GG**	
hnPC12	C **G** C	**Arginine**	**GG**	
hnPC04	C **G** C	**Arginine**	**CG**	Heterozygous
hnPC05	C **G** C	**Arginine**	**CG**	
hnPC11	C **G** C	**Arginine**	**CG**	
hnPC07	N.D	N.D	**CG**	
RP23D	C **C** C	**Proline**	**CC**	Homozygous Proline
RP36E	C **C** C	**Proline**	**CC**	
RP40B	C **C** C	**Proline**	**CC**	
RP68C	C **C** C	**Proline**	**CC**	
RP14D	C **G** C	**Arginine**	**GG**	Homozygous Arginine
RP17B	C **G** C	**Arginine**	**GG**	
RP18F	C **G** C	**Arginine**	**GG**	
RP21D	C **G** C	**Arginine**	**GG**	
RP63A	C **G** C	**Arginine**	**GG**	
RP11D	C **G** C	**Arginine**	**CG**	Heterozygous
RP37A	C **G** C	**Arginine**	**CG**	Heterozygous

N.D, No determined.

### The P72R SNP is associated with prostate cancer risk

**Figure 2:**
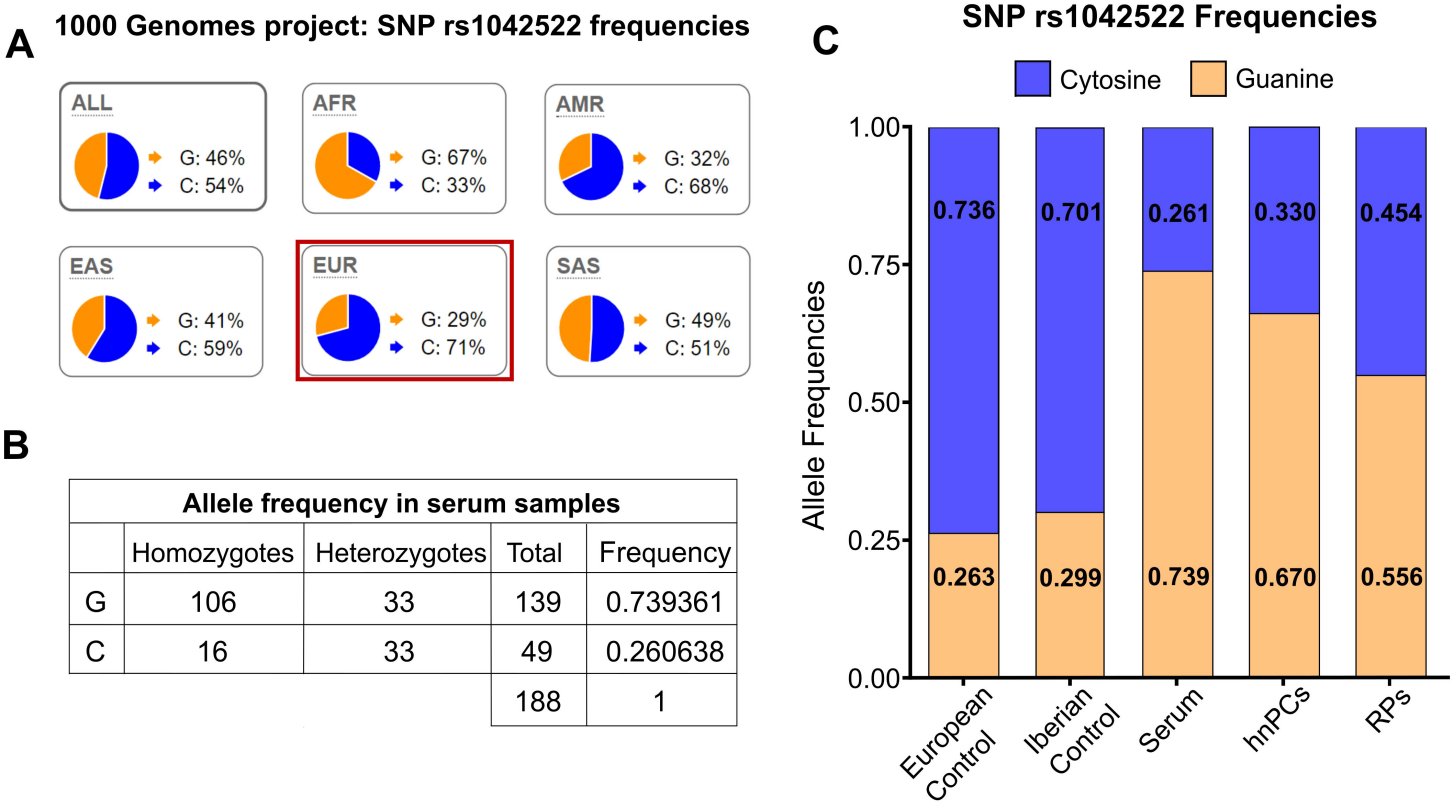
Allele frequencies in the European population and in prostate cancer samples. **(A)** Global frequency of the rs1042522 SNP. The G allele exhibits a lower frequency in all subpopulations except for the African population. Data and images sourced from Ensemble.org. **(B)** rs1042522 frequencies in serum samples from patients with prostate cancer. **(C)** rs1042522 frequencies in primary tumor cultures (hnPCs), serum samples, and radical prostatectomy (RPs) samples compared to those in the control European population and Iberian population.

To validate these results, the serum of 94 patients with prostate cancer was genotyped. P72R is a controversial SNP found at different frequencies across populations worldwide, although the majority of subpopulations exhibit lower frequencies (**Figure 2A**). Among the serum samples, 53.38% (53/94) were homozygous for ARG (hARG), 8.58% (8/94) for PRO (hPRO), and 35.10% (33/94) were heterozygous, whereas the observed frequency of the G allele was 73.9% and the frequency of the C allele was 26.1% (**Figure 2B**). Regarding controls, in the selected European (non-Finnish) non-cancer population from GnomAD, the frequency of the G allele was 26.3%, whereas the frequency of the C allele was 73.6%. Similar frequencies were also found in the Spanish Iberian population (**Figure 2C**). The frequencies in our cohort of serum, hnPC, and RP tissues were then compared with those of the control populations (**Figure 2C**).The results revealed significant differences in the recurrence rate of the P72R SNP in all samples compared with the control population, suggesting an association between the P72R SNP and prostate cancer.

The significance of this association was assessed by contingency analysis (Fisher´s test), which revealed a significant association between the P72R SNP and prostate cancer in all samples analyzed, with a frequency of 73.93% for the G allele and an odds ratio of 7.937 (95% CI 5.37-11.00) for the serum samples (p<0.0001) (**Table 2**).

**Table 2:** Analysis of the association between the rs1042522 variant and prostate cancer.

SAMPLES	Prostate Cancer, n	GnomAD Noncancer, n	Odds Ratio (95% CI)*	P- value*
**Serum samples**				
Arginine (G allele)	139	31021	7.937 (5.73– 11.00)	<0.0001
Proline (C allele)	49	86797		
Total	188	117818		
**hnPCs samples**
Arginine (G allele)	16	31021	5.596 (2.395– 13.080)	<0.0001
Proline (C allele)	8	86797		
Total	24	117818		
**RPs samples**
Arginine (G allele)	12(0.55)	31021	3.358(1.451-7.772)	0.0058
Proline (C allele)	10(0.45)	86797		
Total	22	117818		

Contingency table for the association between the P72R SNP and prostate cancer. The selected European (non-Finnish) noncancer population from GnomAD was used as a control.

*Odds ratio and p values were calculated with the Fisher’s Test.

### The P72R SNP was not significantly associated with high Gleason score

**Figure 3:**
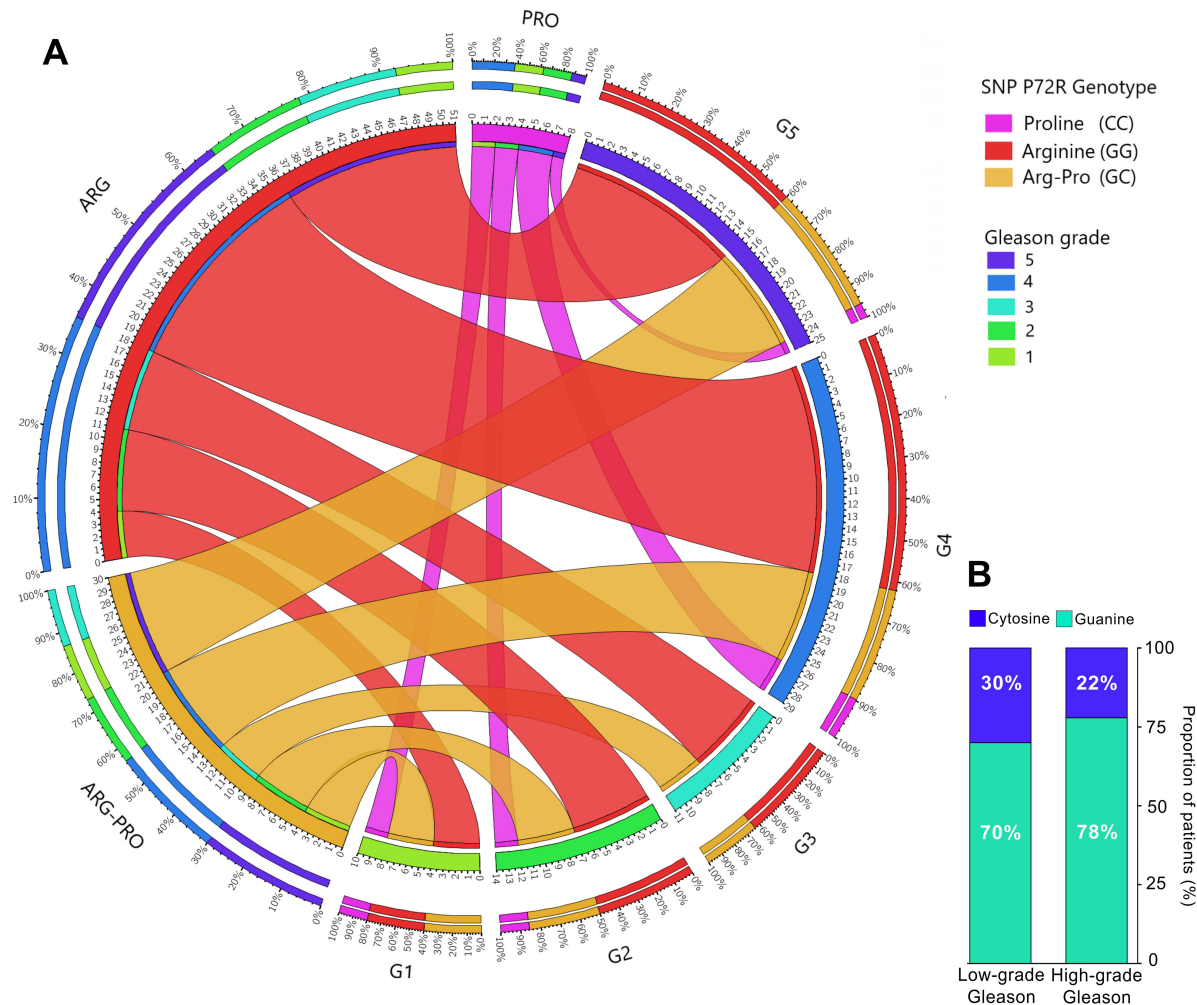
Distribution of the rs1042522 variant in 94 prostate cancer patients according to Gleason grade. **(A)** Circos plot displaying the distribution of the P72R genotypes according to patient Gleason score. **(B)** Distribution of the SNPs according to low Gleason grade (1-3) and high Gleason grade (4-5); contingency analysis of these proportions did not yield significant results. Gleason grade 1: Gleason score ≤6; Gleason grade 2-3: Gleason score = 7; Gleason grade 4: Gleason score = 8; Gleason grade 5: Gleason score ≥9.

The ability of mutant p53 protein to neutralize apoptosis and transform cells in cooperation with EJ-Ras is enhanced when codon 72 encodes ARG (R72) (16). This R72 variant has been reported to bind more efficiently and inactivate PGC-1α (4), a transcriptional target of p53-induced apoptosis in prostate cancer cells, thereby promoting cell invasion and metastasis (4). These observations support our finding that a higher R72 allele frequency is present in more aggressive PCa tumors. To study whether the Gleason Score, or grade (GS) of all genotyped patients was associated with a higher frequency of the R72 allele, we used the Gleason grading group (1 to 5) and divided patients into 2 groups: low-medium aggressive tumors (Gleason grade 1-3), and highly aggressive tumors (Gleason grade 4-5). Patients were genotyped for hARGs, hPROs, or heterozygotes (ARG-PROs). Data from the cohort of 94 serum patients were plotted on a Circos plot according to their Gleason group. **Figure 3A** shows the distribution of patient proportions for each GS. Although a markedly lower proportion of the P72 allele and a markedly greater frequency of the R72 allele were detected in high Gleason tumors, these differences were not statistically significant (p = 0.2867) (**Figures 3A, B**).

## Discussion

The tumor suppressor gene *TP53*, reported to be mutated at a high frequency (53.3%) in aggressive metastatic castration resistant prostate cancer, CRPC (4, 17), was the focus of this investigation. The finding that 9 out of 12 primary tumor cultures from patients with aggressive metastatic PCa had a p53 protein with arginine (CGC) at codon 72, but that only 2 cultures had a proline (CCC) was intriguing. An initial explanation for the enrichment of the R72 variant in hnPCs from cancer tissues was the loss of heterozygosity (LOH), particularly in exon 4, which includes codon 72 of the protein. In fact, loss of the proline (C) allele and preferential retention of the arginine (G) allele have been described in primary tumors and metastatic tissues of squamous cell carcinoma of the vulva (18), head and neck (19), colorectal tumors (20), and lung cancer (21). In addition, a recent meta-analysis using the TCGA pan-cancer database and patients selected for P72R heterozygosity reported that 31% (127/409) of heterozygotes had lost the P72 allele in the corresponding tumor tissue (22), indicating that the G allele is preferentially selected for tumor development, possibly due to enhanced p73-induced apoptosis (16) and the modulation of tumor metabolism by regulating PGC-1α (23). Although proper LOH screening was not performed in our cancer cohort, genotyping analysis of serum samples showed a similar frequency of the R72 variant compared to that of hnPCs and cancer tissues from prostatectomy, suggesting that this variant was obtained through germline, instead of LOH.

The results in serum samples, confirmed the high frequency of the G allele, compared to a control European non-cancer population selected from the GnomAD v2.1, indicating a very significant association (p<0.0001) between this SNP and prostate cancer risk, with an odds ratio of 7.937 (95% CI 5.37-11.00). Several reports have described a similarly significant association between the R72 SNP and the risk of developing cutaneous melanoma, breast cancer, and prostate cancer (24–27). A study on PCa compared 187 Iranian patients with 185 control individuals negative for cancer (27). Although the frequency of the G allele in patients with cancer (65.77%) was not as high as the one reported here (73.93%), the difference was statistically significant compared to the control group (p<0.05).

Notably, several reports have shown controversial results (28–30) or failed to demonstrate a significant association between the rs1042522 variant and the risk of any type of cancer (14, 31–34), mostly because of limited sample size or selection bias. For example, in a colorectal cancer study in the Northern European population, no significant association between the presence of the SNP and the risk of cancer was reported (35), but the healthy control samples selected had a high frequency of the ARG allele (61%), in contrast to the estimated 26% frequency of the (non-cancer) European population from GnomAD v2.1.1. Similar to our findings, one study on prostate cancer reported an association between Pro/Pro and a lower risk of prostate cancer (11). However, several other reports have shown conflicting results (11–14, 36–38), suggesting that an appropriate representation of control samples is necessary for accurate results.

To address potential geographical sub-population biases, we further validated our results by analyzing the 1000 Genomes database, filtered by country, and used the Spanish population as a control. In the control population, we observed that the C allele is present at higher frequencies (0.71) compared to the G allele (0.29), similar to the results obtained with the control non-cancer European population from GnomAD (**Figure 2C**). This indicates that the high frequency of the G allele observed in our serum samples is not attributable to a sub-population effect but it rather reflects its association with aggressive prostate cancer.

These findings underscore the importance of considering population stratification in genetic association studies to avoid potential biases that may skew the results. The use of well-matched control populations is crucial for the accurate interpretation of genetic data. Future studies should continue to account for these variables to ensure the robustness of genetic association findings.

Beyond these conflicting findings regarding ARG frequency at codon 72 of p53, it is crucial to consider the potential impact of this variant on cancer development, although its full understanding is still not clear. Studies in mice have revealed that the presence of the R72 variant is associated with an increased incidence of mammary tumors (39). In addition, this variant is associated with increased phosphorylation of p53 and enhanced transactivation of *CDKN1A* (p21^WAF1^) in response to starvation, with consequent augmented growth arrest and reduced apoptosis, a situation that favors survival (40). Furthermore, *TP53* mutations tended to preferentially occur in the R72 allele. In the presence of mutant *TP53* (*e.g.*, R175H, R273H, and A138V) harboring the R72 SNP, an enhanced capacity for migration, invasion, and metastasis in various cancer cell lines, including PCa, has been reported (23). These observations provide strong evidence that the ARG at codon 72 affects the tumor-suppressive activity of p53.

Although no significant association between the P72R SNP and Gleason score was found in our study, the G allele (R72) was more frequent in patients with a high Gleason score (≥8, group 4-5), suggesting a potential association with more undifferentiated malignant PCa lesions. A few studies have also evaluated the association between this polymorphism and tumor grade and found no significant association with clinical stage or Gleason grade in PCa patients (36, 37, 41, 42). One study revealed a link between the modest influence of this SNP and shorter biochemical recurrence (BCR) after radical prostatectomy (38). Another study in the Japanese population (12) observed a particularly high frequency of the ARG allele in patients with metastases and in those with a high Gleason score (≥8). However, the latter study analyzed the association using the arginine allele as a reference, and the authors calculated the significance level between the Pro/Pro and Pro/Arg genotypes and arginine. As expected, they found no significant differences, as proline did not differ significantly from that in the control group.

To validate and extend our findings, further investigations with a larger sample size and consideration of family history of prostate cancer are needed. Nevertheless, our results suggest that a simple but powerful test (gDNA assessment for the P72R SNP) may be useful for the early identification of patients at risk for aggressive metastatic prostate cancer. Future studies should focus on developing detailed guidelines on how this SNP assessment could be integrated into current prostate cancer screening allowing clinicians to intervene at an early stage.

## Data Availability

Information about the dataset used and/or analyzed during the current study is available in **Supplementary Datasheet 1**. Additional information is available from the corresponding author upon reasonable request.
