# Supplementary material for: Association of the rs1042522 SNP with prostate cancer risk: a study of cancer tissues, primary tumor cultures, and serum samples from a Spanish Caucasian population
Source: Front Oncol. 2024 Aug 13;14:1398411. doi: 10.3389/fonc.2024.1398411 (PMC11347290; doi:10.3389/fonc.2024.1398411)
Supplement: Supplementary file 2 [file Datasheet2.docx]

**Association of the rs1042522 SNP with prostate cancer risk: a study of cancer tissues, primary tumor cultures and serum samples from a European Caucasian population.**

**Supplementary information**

**Table S1. Cycling conditions for melting curve analysis**

| **Phase.** | **Cycles** | **Temperature** | **Hold** | **Ramp rate (°C/s)** |
| --- | --- | --- | --- | --- |
| **Denaturation** | 1 | 95 | 10 min | 4.6 |
| **Cycling** | 45 | 95 | 10 sec | 4.6 |
|  |  | 60 | 10 sec | 2.4 |
|  |  | 72 | 15 sec | 4.6 |
| **Melting** | 1 | 95 | 30 sec | 4.6 |
|  |  | 40 | 2 min | 2.0 |
|  |  | 75 | 0 sec | - |
| **Cooling** | 1 | 40 | 30 sec | 2.0 |

**Table S2. Prostate cancer classification according to Gleason pattern.**

| Patterns* | Score | Group^a^ | Significance |
| --- | --- | --- | --- |
| 1+2, 3+2, 3+3 | ≤ 6 | 1 | Low-grade cancer |
| 3+4 | 7 | 2 | Medium-grade cancer |
| 4+3 | 7 | 3 | Medium-grade cancer |
| 3+5, 4+4, 5+3 | 8 | 4 | High-grade cancer |
| 4+5, 5+4 | 9 | 5 | High-grade cancer |
| 5+5 | 10 | 5 | High-grade cancer |
| *Gleason patterns indicate the grade of tissue differentiation.  ^a^ Detail of the 5 group characteristics:  ^1^Small, uniform glands, well differentiated.  ^2^More stroma between glands, well differentiated.  ^3^Distinctly infiltrative margins, moderately differentiated.  ^4^Irregular masses of neoplastic glands, poorly differentiated.  ^5^Only occasional gland formation, poorly differentiated/anaplastic tissue. | | | |

**Figure S1. SNP P72R in the TP53 gene. A)** The TP53 canonical transcript 201 consists of 11 exons encoding a protein of 393 amino acids, spanning from exon 2 to the 5’ region of exon 11. **B)** The SNP P72R (black arrows) polymorphic variant: Proline (P) is encoded by the triplet CCC, and Arginine (R) is encoded by CGC at position 357 of the coding strand. **C)** Change in the hydrophobic profile of the protein (red arrow) when P is changed to R, a polar-positive amino acid. Figures adapted from Benchling.com. **
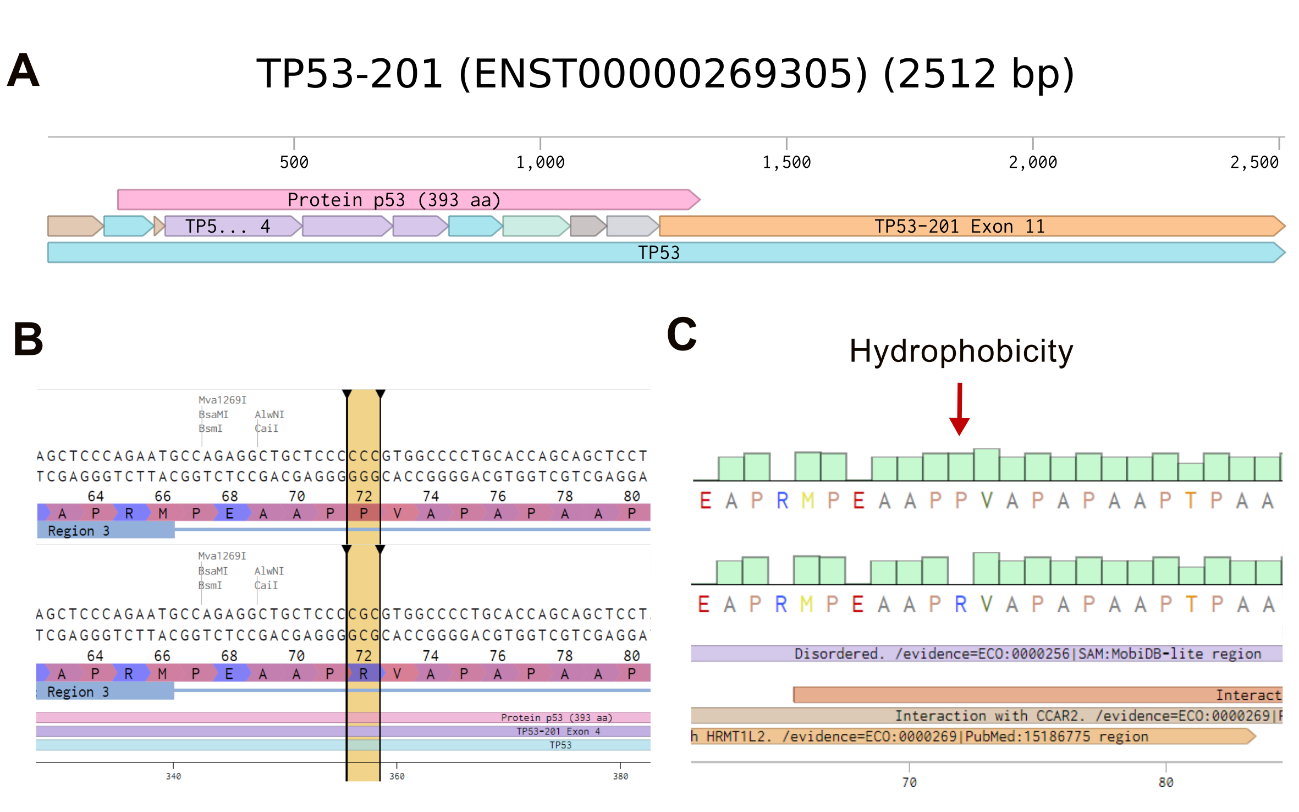
**
